# Cell biology is different in small volumes: endogenous signals shape phenotype of primary hepatocytes cultured in microfluidic channels

**DOI:** 10.1038/srep33980

**Published:** 2016-09-29

**Authors:** Amranul Haque, Pantea Gheibi, Yandong Gao, Elena Foster, Kyung Jin Son, Jungmok You, Gulnaz Stybayeva, Dipali Patel, Alexander Revzin

**Affiliations:** 1 Department of Biomedical Engineering, University of California Davis, CA 95616, USA; 2 Department of Plant and Environmental New Resources, Kyung Hee University, Youngin-si, Gyeonggi-do, South Korea

## Abstract

The approaches for maintaining hepatocytes *in vitro* are aimed at recapitulating aspects of the native liver microenvironment through the use of co-cultures, surface coatings and 3D spheroids. This study highlights the effects of spatial confinement-a less studied component of the *in vivo* microenvironment. We demonstrate that hepatocytes cultured in low-volume microfluidic channels (microchambers) retain differentiated hepatic phenotype for 21 days whereas cells cultured in regular culture plates under identical conditions de-differentiate after 7 days. Careful consideration of nutrient delivery and oxygen tension suggested that these factors could not solely account for enhanced cell function in microchambers. Through a series of experiments involving microfluidic chambers of various heights and inhibition of key molecular pathways, we confirmed that phenotype of hepatocytes in small volumes was shaped by endogenous signals, both hepato-inductive growth factors (GFs) such as hepatocyte growth factor (HGF) and hepato-disruptive GFs such as transforming growth factor (TGF)-β1. Hepatocytes are not generally thought of as significant producers of GFs–this role is typically assigned to nonparenchymal cells of the liver. Our study demonstrates that, in an appropriate microenvironment, hepatocytes produce hepato-inductive and pro-fibrogenic signals at the levels sufficient to shape their phenotype and function.

Primary hepatocytes are commonly used as liver surrogates in bioartificial liver assist devices as well as for toxicology and drug screening^1^,^2^. Upon isolation from the liver and plating in a culture dish these cells rapidly lose an array of hepatic functions as well as their epithelial phenotype, acquiring mesenchymal features and dying within a week. Over the past several decades the liver biology community produced a number of approaches for rescuing the hepatic phenotype^3^,^4^,^5^,^6^,^7^,^8^,^9^. These approaches seek to recapitulate aspects of the *in vivo* microenvironment and include sandwiching hepatocytes between layers of collagen gel or matrigel^6^,^8^,^10^, co-cultures with stromal or other support cells^5^,^11^,^12^,^13^ and forming spheroids^9^,^14^.

Microfluidic devices help minimize the use of cells and reagents while allowing for precise control of the composition and the flow pattern of the media bathing the cells^15^. Several groups have reported the development of microfluidics-based cultures of hepatocytes in order to develop platforms for liver toxicology and tissue engineering applications^16^,^17^,^18^,^19^,^20^. Interestingly, the microfluidic cultures of hepatocytes described to date have relied on continuous perfusion of media and have not reported small-volume cell culture effects of the type discussed here. It is likely that perfusion dilutes endogenous signals in these microfluidic cultures and prevents these signals from reaching threshold concentrations required for affecting hepatic phenotype. In fact, several recent reports, albeit not involving primary hepatocytes, have described flow-induced perturbation of paracrine and autocrine signals inside the microfluidic devices^21^,^22^.

Beebe and colleagues, on the other hand, have pointed to interesting changes in the phenotype of cells cultured in microfluidic channels in the absence of perfusion where transport of molecules is dominated by diffusion^23^,^24^. These reports predicted enhanced effects of autocrine and paracrine signaling in low-volume, diffusion-dominated cell cultures. However, we are not aware of reports from this or other groups describing how diffusion-controlled microfluidic devices may be used to leverage autocrine signaling in maintaining the phenotype of difficult-to-cultures cells such as primary hepatocytes.

Our study began with an observation that, when cultured inside simple microfluidic channels (termed microchambers throughout this paper) in the absence of pumping, primary hepatocytes showed remarkable enhancement in epithelial phenotype compared to cells cultured under identical conditions in 12-well plates (same surface coating, seeding density and media composition). In microscale cultures hepatocytes expressed high levels of E-cadherin, remained polarized, and synthesized albumin at elevated levels over the course of three weeks. Traditionally, such phenotype maintenance in hepatocytes requires collagen gel (or matrigel) sandwich cultures^6^,^8^,^10^, co-cultures with stromal cells^5^,^11^,^12^,^13^ or spheroid cultures^9^,^14^ and has not, to the best of our knowledge, been observed previously in hepatocytes cultured as a monolayer on simple collagen-coated surfaces. In a series of experiments, we demonstrated that phenotype enhancement is the result of endogenous hepato-inductive growth factors (GFs) accumulating inside the microchambers. These observations are interesting because hepatocytes are epithelial cells and are not known for secretion of GFs *in vitro*. We demonstrate for the first time that given correct volume of the culture chamber, these cells are able to turn on a program of autocrine signals and maintain high levels of protein synthesis as well as metabolic activity for three weeks in monolayer cultures. This study is significant on two levels. First, we describe a new method for maintaining differentiated hepatocytes. More significantly, this study brings into focus volume effects as well as accumulation and harnessing of endogenous signals as an important criterion for designing cell culture systems and cellular interactions. We should note that while the present study utilized microfluidic channels housing thousands of hepatocytes, we see no fundamental barriers to increasing the culture area, the number of cells and the number of wells. In fact, efforts are under way in our lab to develop low-volume multi-well plates that mimic traditional biology cultureware while capturing endogenous signals produced by the hepatocytes.

## Results

### Microchambers for culturing hepatocytes

**Figure 1:**
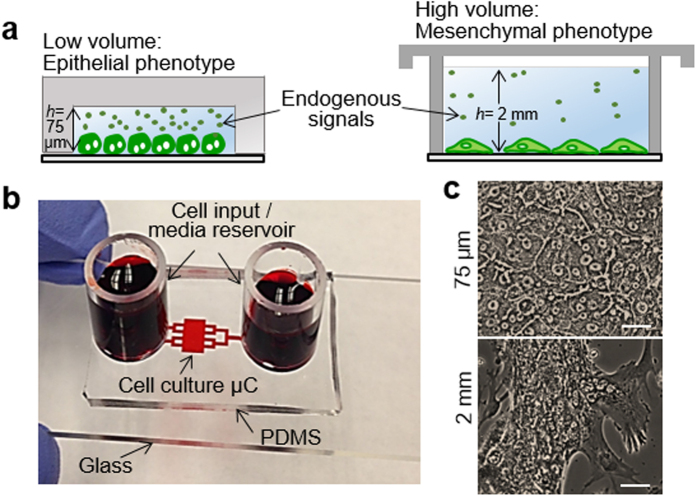
Behavior of hepatocytes in microchambers (μCs). (**a**) Experimental observations-primary hepatocytes retain differentiated phenotype in small volumes but de-differentiate and become mesenchymal in large volumes. The difference in phenotype is connected to the accumulation of endogenous factors in small volumes. *h* denotes the characteristic media height, which is the height of cell culture chamber (75 μm) in case of fluidic device. (**b**) A PDMS-based microdevice perfused with food dye. (**c**) Bright field images of primary hepatocytes after seven days in 12-well plate (2 mm) or inside μC (75 μm). Scale bar is 25 μm.

To harness cell secreted signals, we fabricated a simple microsystem comprised of a cell culture microchamber 3 mm in length, 5 mm in width and 75 μm in height connected by transport channels (600 μm in width and 75 μm in height) to reservoirs at the inlet and outlet holding 500 μl of media (Fig. 1a,b). Protocols were optimized to seed hepatocytes inside the culture microchambers and to minimize attachment of cells at the inlet and outlet reservoirs and in transport channels. As a result, 15,000–20,000 hepatocytes became confined inside a cell culture microchamber. The total volume of the microsystem (500 μl) was dominated by the media reservoirs at the inlet and outlet, however, the immediate volume in which cells were confined was 1 μl (Fig. 1b). The media inside microchambers was changed every 48 h. We characterized the flow pattern by monitoring movement of 1 μm diameter beads and determined the flow rate to be in the range of 0.19 to 0.77 μl/h throughout the culture period. The flow moved back and forth, likely due to the differences in surface tension between inlet and outlet of media reservoirs. The shear stress (<0.000456 dyn/cm^2^) associated with this flow is negligible and is not expected to have a negative effect on hepatocytes which are known to be shear sensitive^16^,^19^,^20^. The flow likely aided in delivery of nutrients to the cells, however, transport in the microchambers was dominated by diffusion. Diffusion-convection modeling was used to predict accumulation of GFs, using HGF as the model factor. The secretion rate for HGF was obtained from ELISA (described later in the paper), and was assumed to remain constant over time and to be the same for both micro- and macro-scale cultures (see Table S1 for modeling parameters). Despite these simplifications, modeling offered valuable insight into geometry-dependent accumulation of secreted factors. As shown in kage^37^. A detailed description of the simulations protocols can be found in the Fig. S1, GFs were expected to accumulate rapidly in the microchambers, reaching 12-fold higher levels compared to standard culture conditions after ~12 h in culture. These higher levels of GFs were expected to be maintained inside the microchambers for 48 h until the next media exchange.

### Evidence for enhanced protein synthesis, metabolic activity and epithelial morphology of hepatocytes cultured in microchamber

**Figure 2:**
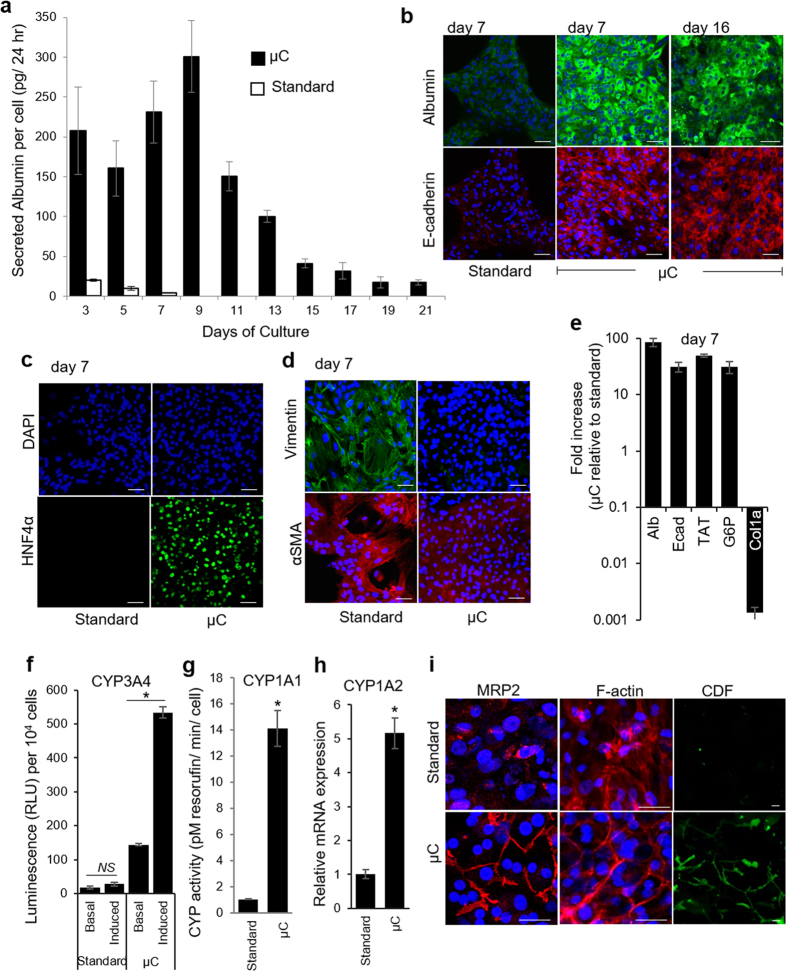
Functionality of primary hepatocytes is maintained inside microchambers (μCs). (**a**) Secretion of albumin by hepatocytes cultured on collagen I-coated glass sides in 12-well plate (standard) and inside μCs. Data shown are mean ± SD (n = 3) for each sample type. (**b**) Immunofluorescence staining of epithelial markers-albumin (green) and E-cadherin (red). (**c**) Immunofluorescence analysis of hepatic marker, HNF4α. (**d**) Immunofluorescence analysis of mesenchymal markers, vimentin (green) and αSMA (red). Nuclei are stained by DAPI (blue). Scale bar = 50 μm. (**e**) Isolated rat primary hepatocytes show enhanced expression of hepatic markers under fluidic conditions. Relative mRNA expression of noted genes were quantitated by qPCR on day 7. Fold increase is relative to values obtained for hepatocytes cultured in standard tissue culture. Abbreviation: Ecad, E-cadherin; Alb, albumin; TAT, tyrosine aminotransferase; G6P, glucose-6-phosphatase; and Col1a, collagen 1a1. (**f**) CYP3A4 activity was measured using the P450-Glo CYP3A4 Assay. The cells were cultured for 5 days as monolayers in μCs and tissue culture plates. On day 7, untreated control cells (basal) were compared to cells treated with the CYP3A inducer dexamethasone (10 μM) for 48 hrs. (**g**) Activity of CYP1A1 analyzed by EROD assay after 7 days of cultures. (**h**) qPCR analysis of CYP1A2 at day 7 in μCs and standard cultureware. Data are shown as mean ± SD for n = 3 samples. *p < 0.05. *NS:* non-significant. (**i**) Distribution of MRP2, intracellular actin filaments (F-actin), and functional bile canaliculi in hepatocytes on day 7. MRP2 and actin are localized to cell-cell contacts in μC. CMFDA live fluorescent images demonstrating functional bile canalicular networks in hepatocytes cultured inside μCs. Carboxyfluorescein (CDF) localizes to bile canaliculi. Scale bar = 25 μm.

Primary hepatocytes cultured in microchambers pre-coated with monomeric collagen I retained typical epithelial morphology–cuboidal cobblestone-like cells with prominent borders and nuclei–for at least three weeks, whereas cells cultured under identical conditions but in 12-well plates de-differentiated, becoming mesenchymal and dying after 7 days (Figs 1c and S2sir>). Markers associated with epithelial (albumin, E-cadherin, and HNF4α) and mesenchymal (αSMA and vimentin) phenotypes were analyzed to better determine the phenotypic state of hepatocytes inside the microchambers. Figure 2a compares albumin synthesis in hepatocytes cultured inside microchambers and standard 12-well plates. As seen from these data, albumin levels in microchambers increase, reaching highest values of 300 pg/cell/ml/24 h by day 9, which is 18-fold higher than reported albumin value for primary rat hepatocytes cultured in collagen gel sandwich^7^. On the other hand, albumin production in 12-well plates decreases precipitously until it bottoms out at day 7. In comparison to standard culture conditions, hepatocytes in microchambers produce ~10 times more albumin at day 3 and ~50 times more albumin at day 7. One can note that, while enhanced in microchambers, albumin production began to decline after day 9. It will be demonstrated later in this study that the decline in hepatic function appears to be due to accumulation of TGF-β1 and that this decline may be prevented by blocking this pro-fibrogenic GF.

Immunofluorescence staining for albumin and E-cadherin confirmed strong expression of these epithelial markers in cells cultured within the microchambers (Fig. 2b). In addition, hepatocytes in microchambers stained strongly for HNF4α, an activator of the epithelial program and repressor of the mesenchymal program^25^, whereas cells in 12-well plates did not produce appreciable quantities of this transcription factor (Fig. 2c). Significantly, the trend in the expression of mesenchymal markers was reversed-cells in standard culture dishes stained strongly for vimentin and αSMA, whereas hepatocytes in microchambers did not (Fig. 2d). RT-PCR analysis of epithelial and mesenchymal markers confirmed ELISA and immunofluorescence data. At day 7, hepatocytes in microchambers expressed epithelial markers albumin, E-cadherin, TAT (tyrosine aminotransferase), and glucose-6-phosphatase (G6P) 85, 30, 49, and 30 fold higher than cells under standard culture conditions (Fig. 2e). At the same time, expression of a mesenchymal marker collagen (I) was four orders of magnitude lower in microchambers compared to 12-well plates.

The activity of cytochrome P450 (CYPs) enzymes, including CYP1A1 and CYP3A4 is very sensitive to culture conditions and provides a reflection of hepatic phenotype^26^. To assess activity of CYP3A4, hepatocytes were challenged for 48 hours with dexamethasone (Dex, 10 μM). A luminescence signal from cells without Dex reflect ‘basal’ level of enzyme activity. As shown in Fig. 2f, hepatocyte challenged with Dex in microchambers exhibited 4 fold higher levels of CYP3A4 at day 7 compared to “basal” state whereas hepatocytes in standard cultures did not show significant CYP3A4 induction compared to “basal” level. Together with significantly higher ‘basal’ levels of CYP3A4 activity in microchamber devices, induced enzyme activity was about 20-fold higher in cells cultured inside microchambers than those in a standard tissue culture plate. Analysis of CYP1A1 induction revealed that hepatocytes in microchambers had 14-fold higher activity than cells in standard tissue culture plates (Fig. 2g). Gene expression analysis of CYP1A2 revealed similar trends, higher expression levels of hepatocytes confined to small volumes of microchambers (Fig. 2h).

Another major indicator of hepatic phenotype is the polarization of the cell membrane into apical domains at the cell-cell contacts and basolateral domains at the cell-matrix sides. The polarization is disrupted by the isolation process and its re-establishment is a major indicator of epithelial phenotype of hepatocytes. Apical polarization involves localization of specific bile salt transporter proteins such as multidrug resistance protein (MRP)2 to the apical domain and formation of bile canalicular networks between cords of adjacent hepatocytes^27^. Immunofluorescent labeling of MRP2 and cytoskeletal F-actin revealed that both of these proteins were localized at cell-cell contact areas, closely resembling *in vivo* polarization (Fig. 2i). In contrast, hepatocytes cultured under standard conditions did not stain for MRP2 and exhibited F-actin stress fibers indicating de-differentiation. The functionality of the canalicular network was examined using 5-(and 6)-carboxy-2′,7′-dichlorofluorescein diacetate (CDF-DA)-a nonfluorescent compound that is hydrolyzed by intracellular esterases into a green fluorescent product. The fluorescence product is transported out of the cell into canalicular space via MRP2 and may be used to visualize the distribution of these transporter proteins. As shown in Fig. 2i, CDF accumulation was more prominent in the cell-cell contact areas of hepatocytes in microchambers suggesting that cells in small volumes had a more functional bile canaliculi network compared to hepatocytes in standard cultures.

Taken together, our data demonstrate dramatic differences between hepatocytes cultured in microchambers and in standard 12-well plates. Cells in microchambers were synthesizing serum proteins, maintaining metabolic activity and expressing markers of polarized/differentiated hepatic phenotype while cells under standard conditions were de-differentiating in a manner typical of hepatocytes cultured under standard conditions. We should note here that some of the markers of hepatic function, including albumin secretion observed in microchambers were comparable to or better than “gold standard” hepatocyte cultures such as collagen gel sandwiches and spheroid cultures^7^,^28^.

### Assessing the effects of oxygen and nutrient delivery on enhanced hepatic phenotype in microchambers

Hepatocyte function may be affected by multiple factors including: substrate mechanical properties^29^,^30^, composition and coating^31^,^32^, cell seeding density^33^, nutrients and media additives (including GFs) as well as oxygenation^1^,^34^. Several of these factors were eliminated by careful experimental design. The cell culture substrate (tissue culture polystyrene, glass, or PDMS), surface coating (monomeric collagen I), cell seeding density and culture media were identical for standard culture dishes and microchambers. Using known rates of hepatic glucose consumption^35^, we modeled glucose delivery to hepatocytes cultured under standard conditions and in microfluidic chambers. This modeling (Fig. S3 and Table S2) revealed that while hepatocytes experience somewhat lower glucose levels in small volume cultures compared to large volume cultures (368 vs 448 mg/dL respectively), these levels were not outside of the glucose concentration range reported as normal for hepatocytes *in vitro* (100 mg/dL and 450 mg/dL)^36^,^37^. Furthermore, given the geometry of our microfluidic chambers where media reservoirs are connected to cell culture chambers via narrow (high resistance) channels, our concern was poor nutrient delivery. This concern was not borne out by modeling and by the fact that hepatocytes remained highly functional in microchambers (see Fig. 2).

Oxygen delivery is another important factor that could conceivably account for differences between standard and small volume cultures of hepatocytes. Differentiated hepatocytes have a high oxygen consumption rate^38^ (Table S3) and design of the microchambers ensured excellent oxygen delivery. The reason for this is that silicone rubber (PDMS) comprising the roof of the microfluidic device is significantly more oxygen permeable than aqueous media (7.9 × 10^−5 ^cm^2^/s vs. 2.8 × 10^−5 ^cm^2^/s). The geometry of a microchamber was such that hepatocytes were separated from the oxygen permeable silicone rubber roof of the device by a 75 μm layer of media. In comparison, hepatocytes under standard culture conditions (e.g. in 12-well plates) reside below a 2 mm layer of media. Utilizing known oxygen consumption rates for hepatocytes^38^, we constructed diffusion-convection-consumption models in COMSOL predicting oxygen tension next to hepatocytes. This modeling suggested that hepatocytes in microchambers were indeed better oxygenated than in standard 12 well plates (127 vs. 51 mmHg) (Table S4). At the same time, the hepatocytes in standard cultures were by no means deprived of oxygen; in fact, oxygen levels in standard culture conditions fell within a normal range for hepatocytes^39^.

However, there remained a possibility that higher oxygen levels in microchambers played a role in enhancing hepatic phenotype. To better assess this role, we sought to create devices where volume above the cell was varied but oxygen tension at the level of the cells was the same. For this purpose, we fabricated microfluidic channels with both the roof and the floor composed of PDMS. These all-PDMS devices were made with either shallower (75 μm) or taller (1 mm) cell culture microchambers. The floor of both microchamber types was composed of ~2 mm PDMS membrane that was thin enough to ensure that diffusion of oxygen from the bottom negated differences in oxygen tension due to the liquid layer above the cells (Table S4).

It should also be noted that flow rates in shallow and taller microchambers were practically identical. This may be explained by the fact that the media reservoirs in these devices (cloning cylinders in Fig. 1a) were connected to cell culture chambers via long and narrow channels with high fluid resistance. Because the resistance was dominated by the narrow microchannels, changing the volume of the cell culture chamber had minimal effect on the total fluid resistance of the microfluidic circuit. For example, the total fluid resistance within the microdevice decreases from 250 Pa.s/mm^3^ to 241 Pa.s/mm^3^ (only 3.2%) as the chamber height increases from 75 μm to 1 mm. Given that flow rate is directly proportional to fluid resistance; flow and perfusion inside the microfluidic circuit were expected to remain unchanged as the volume of culture chamber increased. In summary, microchambers were designed to allow varying volume of media above the cells while keeping oxygenation and flow patterns similar between the devices. The ability to dilute secreted signals without changing flow, oxygen or nutrient levels was a distinct advantage of our system over standard perfusion-based microfluidic channels. In the case of the latter, it is relatively simple to modulate endogenous signals by varying the flow rate but it is difficult to decouple the contributions of autocrine signals from the shear effects as well as from differences in the delivery of oxygen and nutrients.

**Figure 3:**
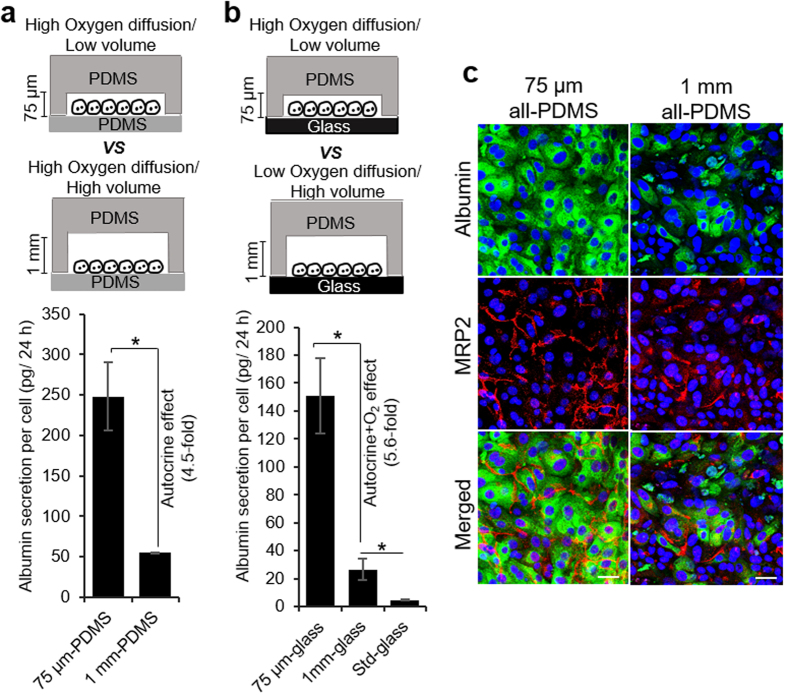
Investigating the effects of oxygen and microchamber volume on phenotype of hepatocytes. Secretion of albumin by hepatocytes cultured on collagen I-coated PDMS substrate (**a**) and glass slides (**b**) in 75 μm and 1 mm tall μCs. Hepatocytes grown for 7 days on collagen I-coated glass slides in 12-well plates were used as control (denoted as standard). The data indicate means ± SD (*n* = 3). *p < 0.05. (**c**) Immunostaining images of albumin (green) and MRP2 (red) in hepatocytes cultured for 7 days in all-PDMS microchambers with variable heights. Nuclei are stained by DAPI (blue). Scale bar = 50 μm.

As shown in Fig. 3a,b, albumin production after 7 days of culture was 4.5 fold higher in 75 μm microchambers than in 1 mm chambers for all-PDMS devices and 5.6 fold higher for PDMS-on-glass microchambers. These data suggest that phenotype enhancement vis-à-vis albumin synthesis was marginally (~20%) affected by oxygenation. Absolute values for albumin synthesis were higher for all-PDMS than for PDMS-on-glass devices, likely due to differences in mechanical properties of the substrates, however, the fold difference in albumin synthesis between taller and shallower microchambers was similar for both scenarios. Immunofluorescent staining of MRP2 and albumin further confirmed that hepatocytes cultured in 75 μm all-PDMS mcirochambers showed better expression of albumin and localization of MRP2 compared to taller microchambers (Fig. 3c).

The results in Fig. 3 may also be viewed from the standpoint of dilution of cell-secreted signals. Diffusion-convection modeling for a given GF (based on HGF secretion rate as described above) pointed to ~12-fold higher concentration of secreted factors in 75 μm chambers compared to 1 mm microchambers (Fig. S1) after 12 h of culture. In light of the fact that oxygen and nutrient delivery were unlikely to be the main contributors to phenotype enhancement, accumulation of endogenous factors and autocrine signaling remained the most likely mechanism of such enhancement.

### Evidence for enhanced production of endogenous GFs in microchambers

**Figure 4:**
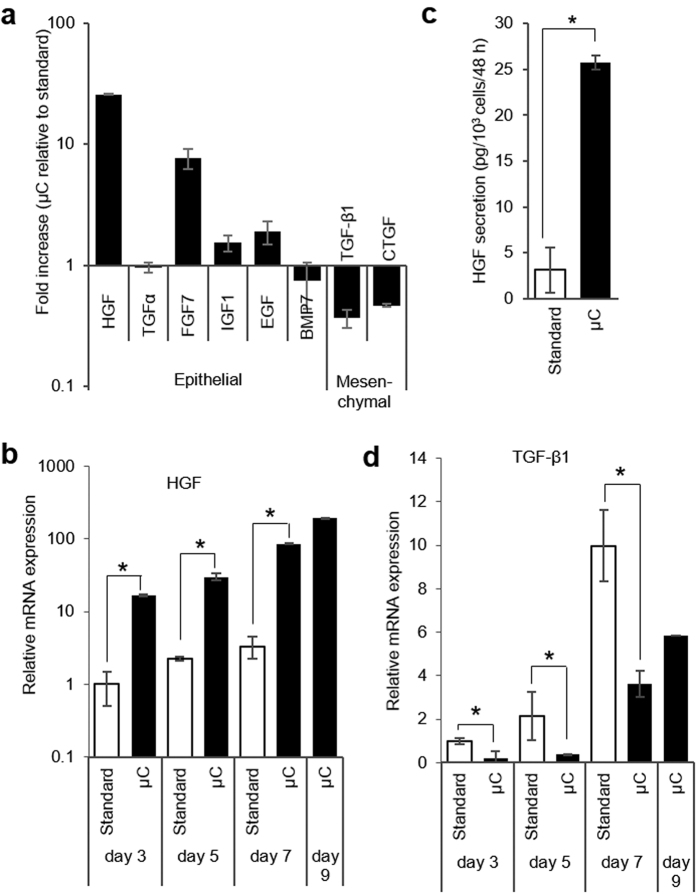
The expression of endogenous growth factors by primary hepatocytes is affected by small volumes of microchambers. (**a**) Quantitative RT-PCR analysis of important liver-relate growth factors at day 7. Fold increase is relative to values obtained for hepatocytes cultured in standard tissue culture. (**b**) Quantitative RT-PCR analysis of HGF expression over 9 days of hepatocyte culture inside μCs and in 12-well plates. (**c**) HGF ELISA of hepatocyte cultured for 7 days μCs and in standard culture plates. (**d**) Quantitative RT-PCR analysis of TGF-β1 inside μCs and in 12-well plates. Data represent the average of 3 biological samples ± SD. *p < 0.05.

While, as noted in the Introduction section, hepatocytes are not known for secretion of GFs, they have been shown to express genes for several ligands including HGF, EGF, IGF1, TGF-α, FGFs, BMPs, CTGF, and TGF-β1^28^,^40^,^41^,^42^,^43^,^44^,^45^. RT-PCR analysis of hepatocyte cultured in microchambers for 7 days revealed upregulated expression of several hepato-inductive GFs (HGF, EGF, IGF1, FGF7) and lower levels of “hepato-disruptive” GFs (TGF-β1, CTGF) compared to cells cultured in standard 12-well plates (Fig. 4a). The differences in gene expression were particularly striking for HGF, the key signaling molecule involved in liver development and regeneration^46^,^47^. Its gene expression was 10 fold higher at day 3 and 20 fold higher at day 7 in microchambers compared to standard cultured (Fig. 4b). ELISA results corroborated RT-PCR analysis, revealing a 7-fold higher level of HGF in microchambers compared to standard cultureware after 7 days of culture (Fig. 4c). The expression of other important hepato-inductive factors, FGF7, EGF, and IGF1, was 8, 2, and 1.5 fold higher in microchambers compared to standard cultures, suggesting that a milieu of endogenous signals contributes to enhanced hepatic phenotype in small volumes (Fig. 4a).

TGF-β1 is a pro-fibrogenic GF associated with liver injury and appearance of mesenchymal phenotype in hepatocytes^48^,^49^. There is evidence pointing to HGF opposing TGF-β signaling and vice versa^50^,^51^. The molecular mechanisms of HGF countering TGF-β remain poorly understood; however, a report by Inoue *et al*. suggested that this interaction is mediated by CTGF^42^. In line with this prior observation, our results (Fig. 4a,d) demonstrate that TGF-β and CTGF transcripts were produced at lower levels by hepatocytes cultured inside microchambers in the presence of high levels of endogenous HGF.

The results presented in Fig. 2, combined with the fact that oxygenation was shown to have only marginal effect on hepatic phenotype in microchambers (Fig. 3a–c), strongly pointed to endogenous signals as the likely reason for enhanced hepatic phenotype. The next section of the paper validates this hypothesis further by manipulating HGF and TGF-β1 signaling in microchamabers.

### Modulation of endogenous signals affects phenotype of hepatocytes in microchambers

**Figure 5:**
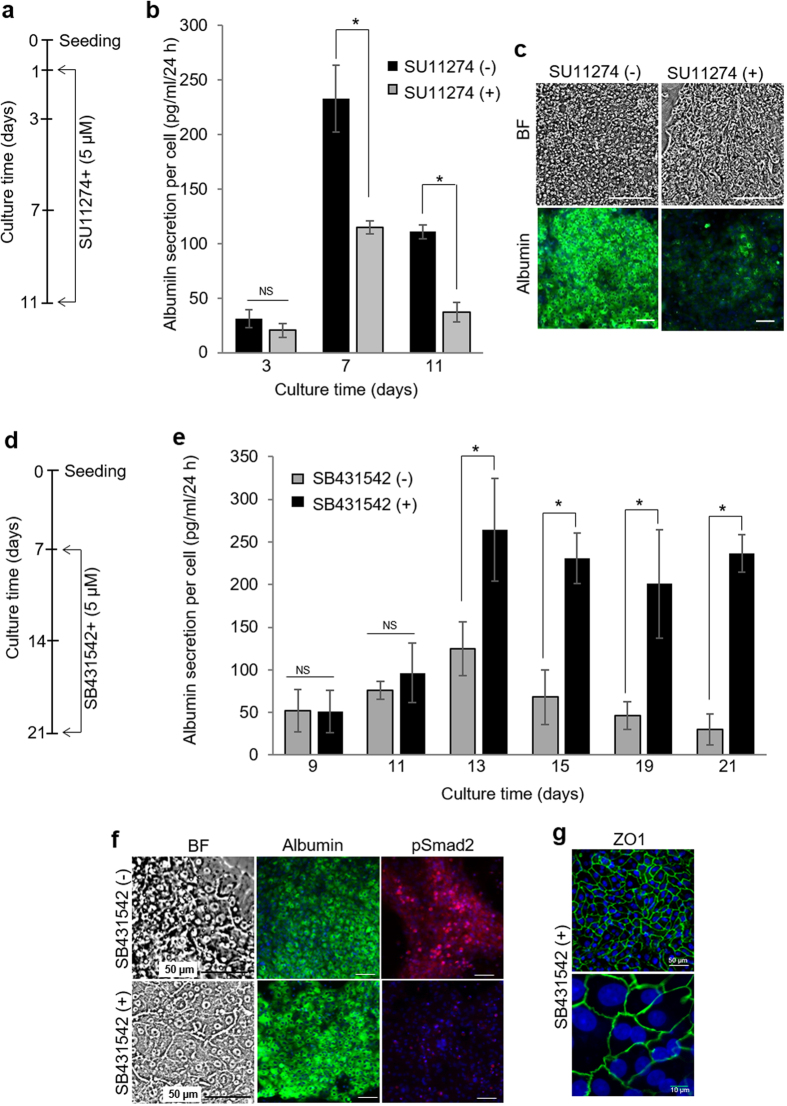
Interfering with HGF and TGF-β1 signaling changes hepatic phenotype in microchambers. (**a**) Hepatocytes were cultured for 24 h inside microchambers and then exposed to 5 μM of c-Met inhibitor SU11274 dissolved in the hepatocyte culture media. (**b**) Inhibition of HGF signaling caused a decrease in albumin secretion by primary hepatocytes, as analyzed by ELISA. (**c**) Bright field and fluorescent images of cells grown in microchambers for 9 days with SU11274 (c-met inhibitor). Cells were stained for albumin (green) and counterstained with DAPI (blue). (**d**) Hepatocytes were cultured for 7 days inside microchambers before introducing 5 μM of SB431542 (*TGF*-β inhibitor) into the culture media. (**e**) In contrast to interference with HGF, inhibition of *TGF*-β signaling enhances albumin synthesis by hepatocytes, as analyzed by ELISA. (**f**) Bright field and fluorescent images of hepatocytes cultured in microchambers for one week in the presence of SB431542 (TGF-β inhibitor). Immunofluorescence staining for albumin (green) and phosphorylated Smad2 (red). Nuclei are counterstained with DAPI (blue). Hepatocytes cultures in microchambers without TGF-β inhibitors were used as control for these experiments. (**g**) ZO1 staining of hepatocytes after two weeks of cultivation inside microchambers in the presence of SB431542. The data indicate means ± SD (*n* = 3). *p < 0.05. *NS*: non-significant. Scale bars are 100 μm unless otherwise mentioned.

To further assess the involvement of endogenous HGF and TGF-β1 in shaping hepatic phenotype in microchambers, we used small molecule inhibitors to interfere with these GFs. First, SU11274 was used to block HGF receptor (c-met) in microfluidic chambers. As shown in Fig. 5a,b, inhibiting HGF signaling in microchambers resulted in 2-fold lower albumin production compared to cells cultured in the absence of inhibitor. Immunofluorescent staining (Fig. 5c) for albumin confirmed the negative effect of c-met inhibition on this hepatic marker. Second, we interfered with TGF-β signaling inside the microchambers. This set of experiments was motivated by observations that albumin synthesis inside microchambers decreased after day 9 in culture (Fig. 2a) while TGF-β1 gene expression went up after 7 days in culture (Fig. 4a,d). It was therefore reasonable to hypothesize that upregulation in endogenous TGF-β1 negatively affected hepatic function in microchambers. Indeed, addition of SB431542, a small molecule inhibiting signaling of TGF-β1 by interfering with activing receptor-like kinases (ALK4, 5, 7)^52^, enhanced hepatic albumin production (Fig. 5d,e). The cells cultured inside the microchambers in the presence of the inhibitor synthesized ~2.5 fold more albumin at day 19 and 10 fold more at day 21 compared to cells cultured under standard conditions. To further prove the importance of TGF-β signaling we performed immunofluorescence staining of phospho (p)-Smad2, a downstream target of the TGF-β/SMAD signaling pathway^48^. As seen from Figs 5f and S4a, hepatocytes in the absence of TGF-β inhibitor stained strongly for pSmad2 and weakly for albumin at day 14 and day 21. Conversely, relatively weak signal for pSmad2 and prominent signal for albumin were observed in the presence of TGF-β inhibitor. Hepatocytes treated with SB431542 did not express mesenchymal marker vimentin after 21 days of culture (Fig. S4a).

In addition to protein synthesis, inhibition of TGF-β affected expression of tight-junction protein ZO-1–a marker of differentiated epithelial phenotype. Immunofluorescent staining for ZO-1 at day 14 and 21 (Figs 5g and S4b) in hepatocytes treated with TGF-β inhibitor SB431542 revealed tight junction and epithelial polarization. These results point to the possibility of interfering with autocrine/paracrine signals inside microchambers to enhance or inhibit hepatic phenotype.

### Can lessons learned from microchamber cultures be translated into standard large volume cultures?

To answer this question, we supplemented the media of hepatocytes cultured in standard 12-well plates with either HGF or TGF-β inhibitor (SB431542). As seen from Fig. S5, hepatocytes cultured in the presence of 50 ng/ml HGF produced 2.2 fold more albumin compared to hepatocytes maintained in 12-well plates in the absence of this GF. Further increasing concentration of exogenous HGF to 100 ng/ml did not result in significant improvement of albumin synthesis. Similarly, hepatocytes cultured in media supplemented with SB431542 synthesized 2.3 fold more albumin than hepatocytes in regular culture media. The results of Fig. S5 demonstrate that while HGF and TGF-β signaling is important in regular cultures, effects of these and other signals are amplified in small volumes of microchambers to the point where exogenous HGF is unnecessary. One can also note that albumin synthesis in microchamber cultures of hepatocytes was ~2.5 times higher than in standard hepatocyte cultures supplemented with either HGF or SB431542. This result may be explained by the fact that the milieu of signals inside microchambers contains multiple hepato-inductive including EGF, IGF1, FGF7 (see Fig. 4a) and possibly other, yet to be determined, factors.

### Microchamber geometry controls hepatic phenotype

**Figure 6:**
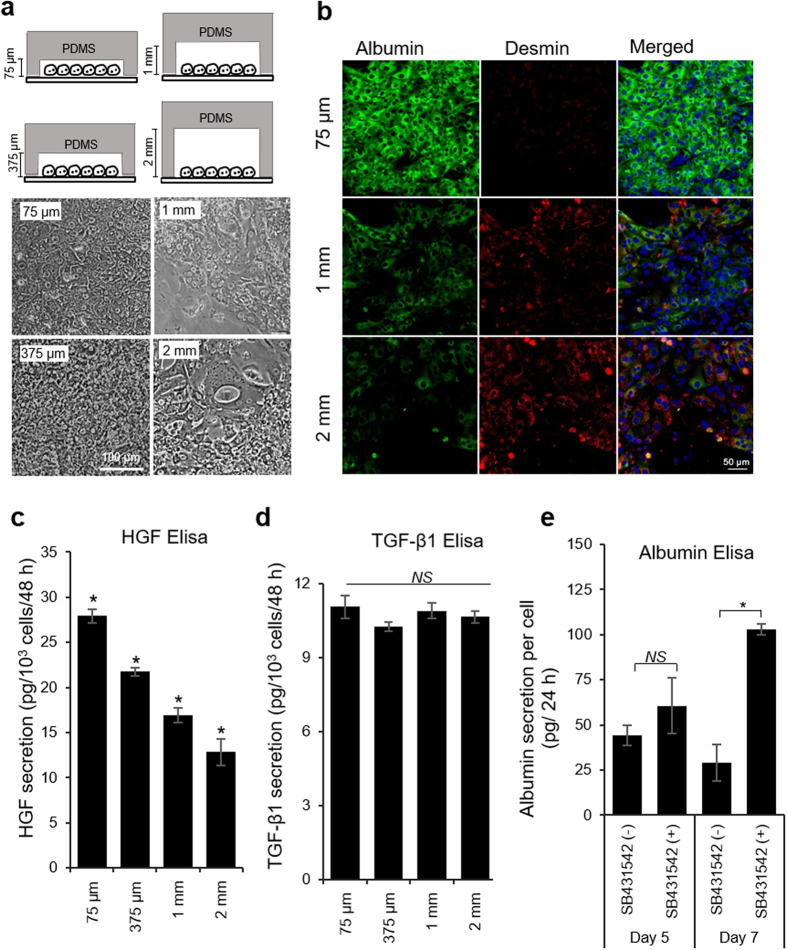
Microchamber geometry affects phenotype of hepatocytes. (**a**) Bright field images of primary hepatocytes cultured on collagen I-coated glass slides for 11 days. (**b**) Immunofluorescence staining for albumin (green) and desmin (red) on 12 days of hepatocyte culture. All these data show that hepatocytes were significantly more functional in shallow microchambers. Nuclei are stained by DAPI (blue). HGF (**c**) and TGF-β1 (**d**) ELISA after 7 days of cultivation inside microchambers of varying height. (**e**) Treatment of primary hepatocytes in standard tissue culture plate with exogenous TGF-β1 inhibitor (SB431542) did not improve hepatic function until 7 days of culture. The data indicate means ± SD (*n* = 3). *p < 0.05. *NS:* non-significant.

Given the central role played by autocrine/paracrine signals in shaping hepatic phenotype in microchambers, we reasoned that changing the height of the microchamber will have the effect of diluting secreted factors. Therefore, we fabricated devices ranging in height from 75 μm to 375 μm, 1 mm and 2 mm so as to increase the local volume from 1 μl to 5 μl, 15 μl and 30 μl, respectively. Similar to the data shown in Fig. 3, increasing the height of the microchamber resulted in loss of epithelial cobblestone morphology and appearance of elongated mesenchymal cells (Fig. 6a). In fact, incremental increase in chamber height caused the phenotype to shift incrementally from epithelial (albumin-expressing cells) to mesenchymal (desmin-expressing cells) (Fig. 6b). Importantly, as highlighted by ELISA results of Fig. 6c, HGF production rate decreased as the local volume of the microchamber increased. These results establish a connection between hepatic phenotype and the levels of endogenous HGF in the microchambers. It is interesting to note that we did not observe differences in TGF-β1 production over the course of 7 days inside microchambers (Fig. 6d). Combined with the data pointing to a decline in albumin synthesis and rise in TGF-β transcipts occurring after ~9 days of cultivation (Figs 2a and 4d), our ELISA results suggest that HGF is the key early signal enhancing hepatic phenotype in small volume cultures and that detrimental effects of TGF-β become evident later on. Furthermore, it is interesting to note that some of the temporal patterns of GF production described for microchambers may be preserved in large volume hepatocyte cultures. Figure 6e highlights that adding TGF-β inhibitor (SB431542) to hepatocytes cultured in a 12 well plate at day 5 of culture does not lead to a significant increase in albumin production while inhibiting TGF-β at day 7 does result in significant enhancement of albumin synthesis.

### Small volume hepatocyte cultures may be combined with biomaterials to further enhance hepatic phenotype

The primary objective of our study was to highlight that hepatic phenotype may be enhanced by confining hepatocytes to small volume cultures. This design criterion may be integrated into other, more established hepatocyte culture systems, for example those utilizing gels. Gel sandwiches comprised of collagen or Matrigel have long been used for cultivation of differentiated hepatocytes^6^,^8^,^10^. Recently, our lab has shown that gels comprised of heparin-PEG copolymer may offer an interesting alternative to natural gels employed previously^53^. The heparin component of the gel can sequester proteins expressing heparin-binding domain (e.g. HGF) and can serve as a depot for their release^53^. In the present study, we coated the floor of the microchamber with heparin-PEG gel, and then deposited collagen I to promote attachment of hepatocytes. We then compared hepatic albumin synthesis in standard 12-well plates coated with monomeric collagen, in collagen gel sandwiches formed in 12-well plates and in microchambers with and without heparin gel coating. It should be noted that in microchambers hepatocytes resided atop and not within the heparin gel coating. As seen from Fig. S6, at day 7 albumin synthesis of hepatocytes cultured in regular (collagen I-coated) microchambers was comparable to that of hepatocytes in collagen gel sandwiches in 12-well plates. Hepatocyte microchambers pre-coated with heparin gel produced 2 times more albumin compared to hepatocytes cultured in collagen gel sandwiches.

## Discussion

Cultivation of primary hepatocytes began over four decades ago^54^,^55^. It was noted early on that hepatocytes cultured *in vitro* lost phenotype and function: protein production, urea synthesis, markers of epithelial phenotype and bile canalicular network. Hepatocytes did not die right away but rather de-differentiated losing epithelial (hepatic) phenotype and becoming more mesenchymal. Over the years, liver biologists identified a number of strategies that recapitulate aspects of the *in vivo* microenvironment to rescue hepatic phenotype in culture. These strategies include gel sandwiches^6^,^8^,^10^ co-cultures^5^,^11^,^12^,^13^ and 3D spheroids^9^,^14^,^56^. Herein, we describe an aspect of *in vivo* microenvironment that has not yet been thoroughly explored for cultivation of hepatocytes, and other cells for that matter–confinement to small volumes. We demonstrate that culturing primary rat hepatocytes in simple microfluidic channels (microchambers) pre-coated with monomeric collagen I, in the absence of perfusion, enhances hepatic phenotype and function to a remarkable extent (Figs 1 and 5). As demonstrated in Fig. 2, hepatocytes in these small volume cultures synthesized albumin, maintained high levels of metabolic activity and expressed numerous markers associated with epithelial hepatic phenotype. The albumin production in microchambers (300 pg/ml/cell/day) was 18 fold higher than that reported for collagen gel sandwiches by Dunn *et al*.^7^ and comparable to some of the best albumin synthesis values reported by Nishikawa *et al*.^57^ for spheroid cultures. It should be noted that function of hepatocytes could be further enhanced by incorporating bioactive heparin gel or stromal cells into microchambers.

We have rigorously investigated mechanisms of hepatic phenotype enhancement in small volume cultures. Most factors were eliminated by carefully matching conditions in microchambers to those in 12-well plates–substrate composition, surface coating, media and cell seeding density were the same for both cases. A diffusion-convection-consumption model was set up in COMSOL to predict glucose concentration at the site of hepatocytes in standard cultures and microchambers (Fig. S3). This model did not reveal distinct differences in glucose levels between the two culture systems. Finally, we addressed the question of oxygen tension as the possible reason for enhanced hepatic phenotype in microchambers. Oxygenation has long been used to enhance hepatic function *in vitro*^38^,^57^. To negate oxygen effects, we fabricated microchambers of different height (75 μm and 1 mm) where both the roof and the floor were comprised of PDMS. Because PDMS is highly oxygen permeable, these devices allowed us to modulate the volume above the hepatocytes while keeping oxygen levels the same. Hepatic function was enhanced 4.5 times in all-PDMS devices suggesting that it is the accumulation of endogenous signals that enhances hepatic phenotype in microchambers and that cell-to-volume ratio is the key design criterion affecting cellular phenotype. Simple diffusion-convection modeling (Fig. S1) reveals that secreted signals, for example HGF, accumulate rapidly in microchambers, reaching 12 fold higher concentrations compared to standard 12-well plates after 12 h of culture. In fact, multiple hepato-inductive signals (HGF, EGF, IGF, FGF7) were upregulated inside microchamber cultures while key hepato-disruptive signals (TGF-β1 and CTGF) were downregulated compared to 12-well plates.

We should note that hepatocytes have been reported to express GFs and their cognate receptors^45^,^58^,^59^; however, to the best of our knowledge, these cells have not been shown capable of producing endogenous signals in sufficient amounts to regulate hepatic phenotype and function in long term cultures. We believe that standard large volume cell cultures drown out effects of endogenous signals whereas cultivation in confined volumes brings these signals to the fore. It is also conceivable that enhanced accumulation of secreted signals plays an important role in other successful methods for culturing hepatocytes-gel sandwich and 3D spheroid cultures. In fact, some evidence of autocrine signaling in hepatocytes spheroid cultures has recently been demonstrated by Griffith and colleagues^28^; however, 3D geometry makes it difficult to decouple autocrine signals from ECM effects.

Diffusing growth factor signals are important in development/tissue patterning^60^,^61^ and also in adult cells–for example branching morphogenesis of mammary epithelial cells^62^. It is therefore reasonable to propose that autocrine signals play an important role in regulating phenotype of hepatic epithelial cells. Our study has practical significance in offering a new, simple way of maintaining functionally competent hepatocytes. However, more importantly, it paints a picture of hepatocytes as active participants in regulating and shaping their microenvironment via autocrine and paracrine signaling. This picture aligns well with some of the recent studies calling for increased emphasis on hepatocytes as perpetrators of liver injury and not just innocent bystanders^63^.

Beyond hepatocytes, the volume effects have recently been observed by us in cancer cells and embryonic stem cells. While cancer cells upregulated endogenous fibroblast growth factor (FGF)-2 and became spontaneously resistant to a kinase inhibitor drug vemurafenib when cultured inside microchambers^64^, mouse embryonic stem cells maintained pluripotency inside microchambers by producing endogenous leukemia inhibitory factor (LIF)^65^. The fact that very different cell types ranging from highly differentiated primary hepatocytes to cancer cells to pluripotent stem cells turn on autocrine signals inside small volumes of microchambers points to the generality of the concept. Therefore, the present study may be viewed as part of emerging evidence that culturing cells in small volumes and confined spaces dramatically affects phenotype through accumulation of higher, more physiological concentrations of secreted ligands and triggering of autocrine signals.

## Materials and Methods

### Fabrication of microchambers

#### Microfluidic single-chamber device

Soft lithography was used to fabricate the microfluidic devices^66^. The patterned SU-8 mold was fabricated by utilizing a silicon wafer (University Wafer), SU-8 2050 (Microchem Corp) and a designed photomask with the microchambers’ features (CAD/Art Services). The single layer of polydimethylsiloxane (PDMS; Dow Corning) was made by pouring a mixture of 10:1 ratio of the base to curing agent onto the SU-8 mold, followed by a degassing step for 30 min before baking at 70 °C for 80 min. The solidified PDMS was then removed from the master mold and a metal puncher was used to create holes for the inlet and outlet of the channel. After oxygen plasma treatment, the PDMS was irreversibly bounded to 75 mm × 25 mm glass slide. Cloning cylinders (10 mm; Fisher Scientific) were attached atop the inlet and outlet of microchannel to serve as media reservoirs. Typical microchamber (length × width × height of 5 mm × 3 mm × 75 μm) is shown in Fig. 1a.

#### Microfluidic single-chamber device with variable heights

In order to design microchambers with variable height, glass slides with known thicknesses (300 μm, 1 mm, and 2 mm) were cut into small rectangles and irreversibly bounded to the 75 μm SU8 mold. The width and the length of the rectangular glass pieces (5 mm × 3 mm) were kept identical to that of actual 75 μm culture chamber in order to increase the dead volume above the cells without disturbing other physical parameters. Subsequently, PDMS prepolymer was poured and cured over the mold to create microchambers of desired height. Noteworthy, the narrow transport channels (600 μm width and 75 μm height) among all devices in the inlets and outlets were kept same to maintain a constant media flow between devices with variable heights.

#### Microfluidic all-PDMS single-chamber device

All-PDMS devices were fabricated by half-baking technique, where both the roof and the floor were comprised of PDMS. We also fabricated all-PDMS microchambers of different heights following similar techniques mentioned above. These all-PDMS devices were made with either shallower (75 μm) or taller (1 mm) cell culture microchambers. The floor of both microchamber types was composed of ~2 mm PDMS membrane.

### Hepatocyte cultures in microchambers

All animal experiments were performed with the approval of the IACUC (Institutional Animal Care and Use Committee) of UC Davis (University of California, Davis) and in accordance with the Ethical Guidelines for Animal Experimentation of UC Davis. Adult female Lewis rats weighing 125–200 g were purchased from Charles River Laboratories (Boston, MA). Typically, 100 to 200 million primary rat hepatocytes were isolated with viability >90% as determined by Trypan Blue exclusion. Primary hepatocytes were maintained in Dulbecco’s Modified Eagle Medium (DMEM; Gibco, 11995) supplemented with 10% (vol/vol) FBS (Invitrogen), 0.5 U/ml insulin (Novolin N), 20 ng/ml epidermal growth factors (EGF; Invitrogen), 7 ng/ml glucagon (Sigma), 7.5 μg/ml hydrocortisone sodium succinate (Prizer), and 1% (vol/vol) penicillin-streptomycin (Invitrogen) as described in literature^53^. The microchannels were coated with 0.2 mg/ml of collagen type I (BD Biosciences) solution for 1 h at 37 °C, followed by a PBS wash prior to cell seeding. For 75 μm height chamber, hepatocytes were first suspended in culture medium at a concentration of 4 × 10^6 ^cells/ml. With an initial flow generated by 50 μl of cell suspension in the inlet, we were able to maintain the seeding density between 15,000 to 20,000 cells (100,000 to 133,000 cells/cm^2^) inside 75 μm height chambers. As has been explained in the microchamber fabrication section, we were able to design our culture chambers in such a manner that increasing chamber height does not affect perfusion rate. Increasing microchamber height from the original 75 μm to 375 μm to 1 mm to 2 mm would result in an increase in local volume by 5-, 15- and 30-fold respectively. Since, the cell growth area (3 mm × 5 mm) in devices with different heights were kept constant, in order to maintain the same cell density (100,000–133,000 cells/cm^2^) we diluted initial cell suspension (4 × 10^6 ^cells/ml) by a factor of 5, 15, and 30 to maintain the number of cells constant among devices with different geometry. After seeding, cells were removed from the inlet and outlet and 500 μl of media was added to each reservoir. For standard culture, glass slides (1.2 cm × 1.2 cm) coated with collagen type I were placed in 12-well tissue culture plates. Primary hepatocytes were seeded at a density of 100,000 cells/cm^2^ for 60 min at 37 °C. The samples were then washed with PBS to remove unbound hepatocytes and 1 ml of fresh media was added to each sample well. Media was changed every 48 hrs, except for the first medium change, which was done at 24 hrs after plating and was used to introduce any added growth factors and inhibitors used for control experiments. For example, following 24 hrs of plating, the hepatocytes were cultured for 6 days in standard hepatocyte media, mentioned above, containing 50 and 100 ng/ml human HGF (Invitrogen), 5 μM c-met inhibitor (SU11274; Sigma), or 5 μM TGF-β inhibitor (SB431542; Sigma).

### Hepatocyte cultures in collagen sandwich and heparin gels

The protocols for fabricating collagen gel sandwiches and heparin hydrogel have been described elsewhere^67^. Briefly, for collagen gel sandwiches, 1.2 cm × 1.2 cm glass slides were coated with 30 μl/cm^2^ of an ice-cold solution of 1 mg/ml collagen I. The samples were incubated at 37 °C for 60 min to induce collagen gelation. Collagen gel-coated slides (mentioned above) were placed in 12-well plates for cell culture. Each well received 5 × 10^5^ primary hepatocytes in hepatocyte culture medium, incubated for 2 h and then washed thrice with PBS. A second layer of collagen gel was then placed on top of the cells one day after seeding. Cells were cultured for 7 days in order to monitor the secretion of albumin. Heparin-based hydrogels (5% w/v) were prepared on silane-coated glass substrate by UV-initiated thiol-ene polymerization of thiolated heparin (Hep-SH) and diacrylated poly (ethylene glycol) (PEG-DA), as reported previously^67^. To form cross-linked heparin gel the slides were exposed to 365 nm, 180 mW/cm^2^ UV light using an OmniCure series 1000 light source (EXFO) for 5 sec. Heparin-coated glass slides were placed in 12-well plates or used for fabricating microchamber devices. The substrates were then incubated with 0.2 mg/ml of collagen type I for 1 h, and washed in PBS.

### Immunostaining and live cell fluorescence imaging

Microchambers were washed with PBS prior to the fixation step. Cells were fixed in a solution of 4% paraformaldehyde (Electron Microscopy sciences) +0.2% Triton-X100 (Invitrogen) in PBS (Invitrogen) for 15 min. After washing with PBS, samples were incubated in blocking solution (1% bovine serum albumin (BSA) in PBS) for 90 min. The samples were then incubated with primary antibody solution for 90 min, followed by washing with PBS for 5 min. Finally, the samples were incubated with a mixture of secondary antibody and DAPI (Invitrogen) for 60 min and washed with PBS for 5 min before taking images. All staining experiment was carried out at room temperature. The primary antibodies used were: sheep anti-rat albumin (1:100; Bethyl lab Inc.), mouse anti-E-cadherin (1:50; BD Transduction Lab.), rabbit anti-vimentin (1:100; Santa Cruz), rabbit anti-hepatocyte nuclear factor (HNF)-4α (1:100; Santa Cruz), rabbit anti-α-smooth muscle actin (αSMA, 1:100; Abcam), mouse anti-desmin (1:20; Sigma), rabbit anti-multidrug resistance associated protein (MRP)-2 (1:100; Sigma), rabbit anti-ZO1 (1:100; Invitrogen), and rabbit anti-phospho-Smad2 (ser465/467)/Smad3 (Ser423/425) (1:200; Cell Signaling). The secondary antibodies used were: Alexa-488 donkey anti-sheep IgG, Alexa-488 donkey anti-rabbit IgG, Alexa-546 donkey anti-mouse IgG, and Alexa-546 donkey anti-rabbit IgG. Secondary antibodies were purchased from Invitrogen and diluted 1:1000. For actin visualization, a 1:40 dilution of rhodamine-conjugated phalloidin (Invitrogen) was added to fixed and permeabilized samples for 30 minutes. Stained cells were visualized and imaged using a laser scanning confocal microscope (LSM700, Carl Zeiss, Jena, Germany).

For visualization of functional bile canalicular networks, samples were rinsed twice with warm regular Hank’s balanced salt solution (HBSS) after which 3 μM CMFDA (Invitrogen) in HBSS was added to each well. After a 15–20 min incubation, the cells were rinsed three times with cold HBSS. 5-(and 6)-carboxy-20′,70′-dichlorofluorescein (CF) accumulation in the bile canaliculi was visualized by fluorescent microscopy using a Nikon microscope at day 7.

### Analysis of hepatic function and gene expression

Albumin (Bethyl Laboratories), HGF (R&D systems), and TGF-β1 (R&D systems) secretion was assessed using commercial ELISA assay. ELISA values were normalized to the cell number. ImageJ was used to count the cell number from DAPI stained images^68^.

Cytochrome P450 function was evaluated based on the ethoxyresorufin o-deethylase (EROD) activity, which is mainly catalyzed by the isoenzyme CYP4501A1. The activity was measured as the rate of resorufin formation from the ethoxyresorufin substrate. In this assay phenol red-free DMEM (Corning) and Earl’s balanced salt solution (EBSS; Gibco) was used for hepatocyte culture medium to avoid interference with fluorescence measurements. Briefly, 2 μM of the inducer, 3-methylcholanthrene (Santa Cruz), was added to the culture medium 48 hr before the assay. The substrate solution, containing 8 μM ethoxyresorufin (Sigma) and 90 μM dicoumarol (Santa Cruz) in EBSS was added to each inlet or well after washing 2 times with PBS. After 5, 10, 20, 35, 50 min of incubation 20 μl of substrate solution was taken from each well in triplicates and dispensed into a 96-well plate. Fluorescence intensity was measured with a Tecan Infinite F50 Microplate Reader (excitation at 530 nm wavelength, emission at 580 nm wavelength) and converted to a resorufin concentration based on a standard curve ranging from 0 to 1,000 nM.

CYP3A4 activity of primary rat hepatocytes was measured using the P450-Glo CYP3A4 Assay (Promega). The treatment was initiated on day 5 by replacing hepatocyte culture medium with medium containing 10 μM dexamethasone or vehicle (hepatocyte culture media only). After 48 hrs of treatment (on day 7), the treatment medium was replaced with medium containing 50 μM Luciferin-PFBE. Four hours later, CYP3A4-dependent conversion of Luciferin-PFBE to luciferin was determined by combining a sample of medium in a separate 96-well plate with luciferin detection reagent according to manufacturer’s instruction. Luminescence was measured on a Tecan Infinite F50 Microplate Reader. Last, cell number was determined by counting DAPI stained nucleus using imageJ software and relative luminescence units (RLU) from the P450-Glo™ Assay were normalized to cell number.

For quantitative RT-PCR analysis, cells were collected from microchambers by treatment with 100 μl 0.05% trypsin-EDTA (Gibco) for 10 min at 37 °C. The dissociated cells were harvested at the outlets. Total RNA was harvested, reverse transcribed into cDNA and subsequently used as template for PCR according to manufacturer’s instruction (Roche). All PCR reactions were done in duplicates using universal SYBR Green master (Roche). The relative expression level of each gene was calculated using the comparative threshold cycle (Ct) method with GAPDH as the housekeeping gene. The number of samples (culture surfaces) used for statistical analysis of PCR data was n = 3 for all conditions. Primers used for this quantitative real-time PCR analysis are listed in Table S5.

### Characterizing fluid flow in microchambers

Flow pattern and flow rate inside microchambers were assessed by monitoring movement of beads. Prior to experiments, both the fluoro-max red fluorescent particles (1 μm diameter; Fisher Scientific) and microfluidic channels were blocked with 1% BSA for 1 hr at room temperature. The particles were dissolved in PBS with 20% OptiPerp (Sigma) and 1% BSA to estimate the flow rate of media in mirochamber devices. To mimic conditions of media exchange, 250 μl of media with tracing beads was added into each cloning cylinder and movement of beads was monitored for 24 hr using time lapse microscopy.

### Statistical analysis

Experiments were repeated at least 3 times with duplicate samples for each condition. Data from representative experiments are presented. All error bars represent standard deviation (SD). Student *t*-test was used for statistical analysis. Differences were considered to be statistically significant at p < 0.05.

## Additional Information

**How to cite this article**: Haque, A. *et al*. Cell biology is different in small volumes: endogenous signals shape phenotype of primary hepatocytes cultured in microfluidic channels. *Sci. Rep.* **6**, 33980; doi: 10.1038/srep33980 (2016).

## Supplementary Material

Supplementary Information
