# Supplementary material for: Cell biology is different in small volumes: endogenous signals shape phenotype of primary hepatocytes cultured in microfluidic channels
Source: Sci Rep. 2016 Sep 29;6:33980. doi: 10.1038/srep33980 (PMC5041105; doi:10.1038/srep33980)
Supplement: Supplementary Information [file srep33980-s1.pdf]

SUPPORTING INFORMATION

Biology is different in small volumes: endogenous signals shape phenotype of primary hepatocytes cultured in microfluidic channels

Amranul Haque, Pantea Gheibi, Yandong Gao, Elena Foster, Kyung Jin Son, Jungmok You, Gulnaz Stybayeva, Dipali Patel, & Alexander Revzin

SI Materials and Methods

Modeling of GF accumulation, oxygen transport and glucose consumption in microchambers. In order to estimate the local concentration of endogenous HGF, oxygen tension and sufficient supply of glucose inside microchambers and the standard culture system, numerical simulations were performed using COMSOL Multiphysics 4.3 software (COMSOL Inc., Los Angeles, CA). For simplification of the modeling, the rates of HGF secretion, oxygen consumption and glucose consumption were assumed constant for all cell culture conditions. The secretion rate for HGF was obtained from ELISA (Fig. 4c), whereas glucose and oxygen consumption rates were previously reported in the literature [1] for oxygen, and for glucose from Nyberg et al. [2]. All parameters utilized for the numerical simulations are provided in Supplementary Table 1, 2 and 3.

Local concentration of endogenous HGF:

The following equation was used for modeling HGF concentration:

$$\frac{\partial c_{HGF}}{\partial t} = \nabla \cdot (D_{HGF} \nabla c_{HGF}) - u \cdot \nabla c_{HGF} + R_{HGF}$$

c_{HGF} is HGF concentration (nM), D_{HGF} is the diffusivity of HGF (cm^2/s) in medium and u is the flow velocity (μm/s), which was estimated experimentally. We set up the velocity of the flow (medium) inside microchambers by exploiting interpolation function in COMSOL.

Lastly, R_{HGF} was set as the endogenous HGF secretion by cells, which can be described as follows:

$$R_{HGF} = \sigma_{sec} \cdot \rho_{cell}$$

where σ_{sec} is the HGF secretion rate (mM/h/cell) and ρ_{cell} is the cell density.

Oxygen tension levels at the cell surface:

Oxygen tension was modeled using the following equation:

$$\frac{\partial c_{O_2}}{\partial t} = \nabla \cdot \left(D_i \nabla c_{O_2}\right) - u \cdot \nabla c_{O_2} + R_i$$

c_{O2} is oxygen concentration (mol/L), u is the average velocity and D_i is the diffusivity of oxygen (cm^2/s) in cell, medium and PDMS membrane. R_i is 0 for medium and PDMS and for cell, R_i is the oxygen consumption of cells followed by Michaelis-Menten kinetics.

$$R_i = -\frac{V_{max} \rho_{cell} c_{O_2}}{K_m + c_{O_2}}$$

V_{max} is the maximum respiration rate per cell (mol/cell/s), K_m is the Michaelis-Menten constants (μM). The initial oxygen concentrations are set to be 220 μM for cell, 0 μM for medium, and 250 μM for PDMS membrane [3].

Moreover, the following assumptions were made:

1) there exist a continuous pO_2 and mass flux of oxygen at interfaces between cell and medium or medium and PDMS; 2) the pO_2 on PDMS surfaces is constant (pO_2 at PDMS surface = $P_g \times s_{PDMS}$ where s_{PDMS} is oxygen solubility in PDMS); 3) the bottom surface (glass) is oxygen-impermeable.

Glucose supply:

The equation used for modeling glucose supply is

$$\frac{\partial c_{glucose}}{\partial t} = \nabla \cdot \left(D_{glucose} \nabla c_{glucose}\right) - u \cdot \nabla c_{glucose} + R_{glucose}$$

In the above equation $c_{glucose}$ is glucose molar concentration, $D_{glucose}$ is the diffusivity of glucose (cm^2/s) in medium and u is the flow velocity (μm/s), which is estimated experimentally. Again, interpolation function in COMSOL was exploited here to set up the velocity of flow (medium) inside microchambers. $R_{glucose}$ is the glucose consumption of cells, which can be described as follows:

$$R_{glucose} = \sigma_{con} \cdot \rho_{cell}$$

where σ_{con} is the glucose consumption rate (nmol/s/cell) and ρ_{cell} is the cell surface density ($/mm^2$).

Supplementary Figures

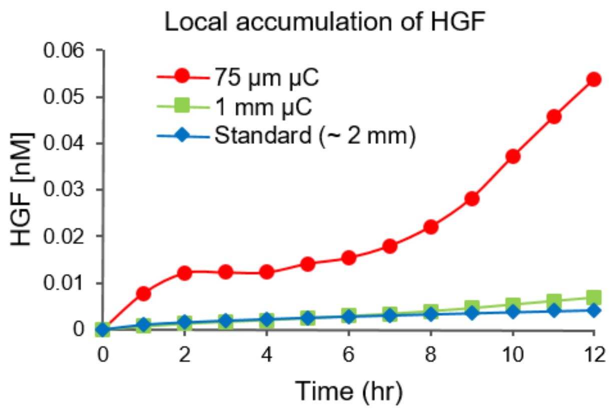

Fig. S1. Simulation of endogenous factor (HGF) concentration for primary hepatocytes cultured inside μCs, compared to standard tissue culture plate.

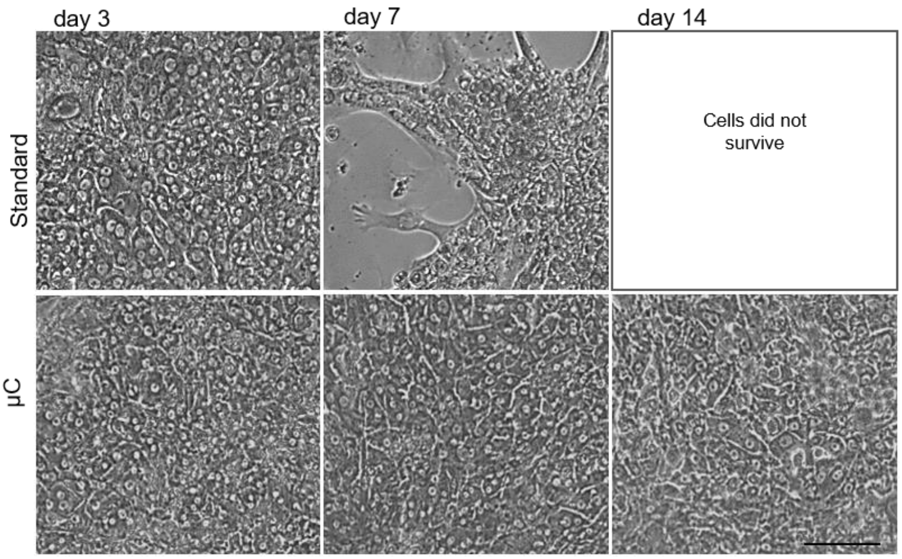

Fig. S2. Bright field images of primary hepatocytes cultured on collagen I-coated glass slides for indicated number of days in standard tissue culture and inside microchambers (μCs). Scale bar is 100 μm.

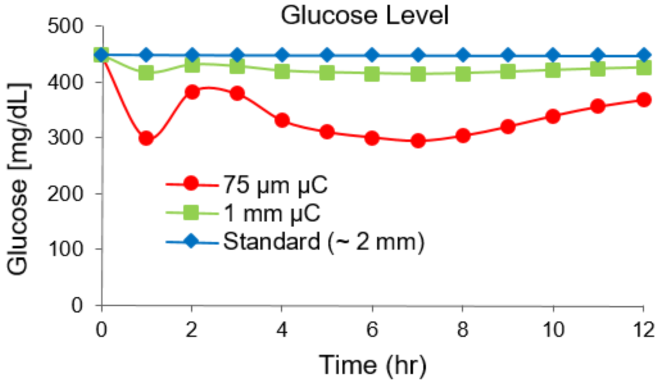

Fig. S3. Simulation of glucose concentration for primary hepatocytes cultured inside microchambers (μCs) with variable chamber heights, compared to standard tissue culture plate.

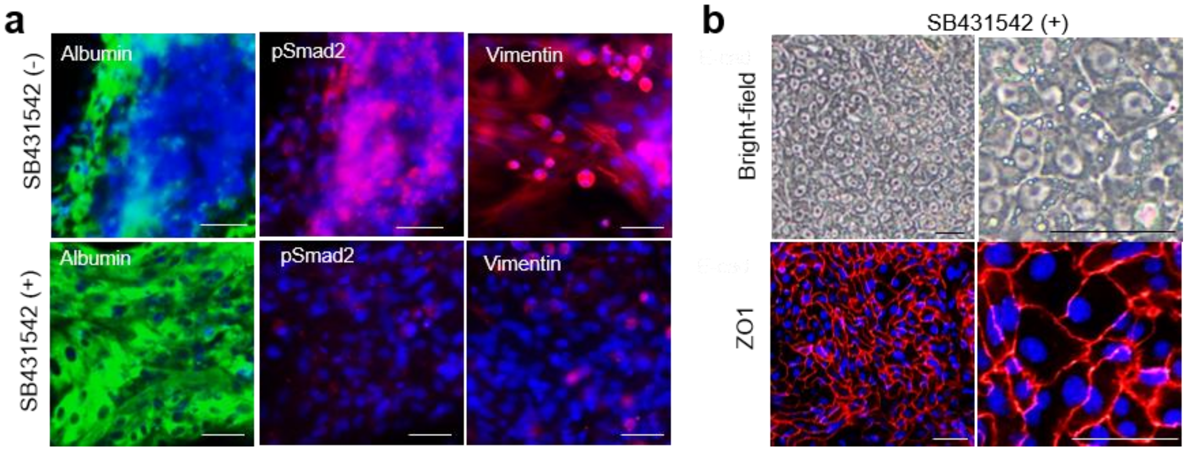

Fig. S4. TGF-β1 inhibitor (SB431542)-treated primary hepatocytes cultured inside microchambers maintained hepatic function and morphology over three weeks. (a) Fluorescent images of primary hepatocytes grown in microchambers for two weeks with SB431542 (total culture period: 3 weeks). The cells were stained for albumin, phospho-Smad2, and vimentin. (b) Bright field images and fluorescent staining of ZO1 demonstrating defined membrane boundaries reflecting normal development of tight junctions and epithelial polarization. Nuclei are stained with DAPI. Scale bar = 50 μm.

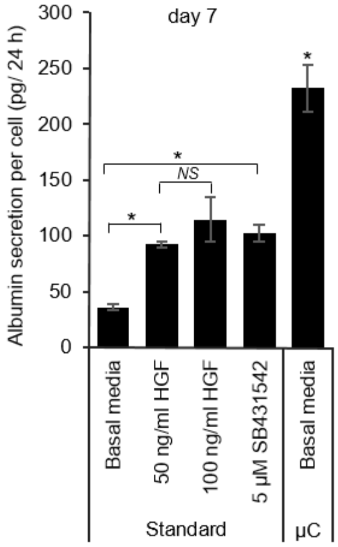

Fig. S5. Effect of exogenous recombinant HGF and TGF-β1 inhibitor (SB431542) on hepatic function. Treatment of primary hepatocytes in standard tissue culture plate with exogenous HGF protein and TGF-β1 inhibitor (SB431542) for 7 days enhanced production of albumin. The data indicate means ± SD ($n = 3$). *:$p < 0.05$. *NS*: non-significant. µC: microchamber.

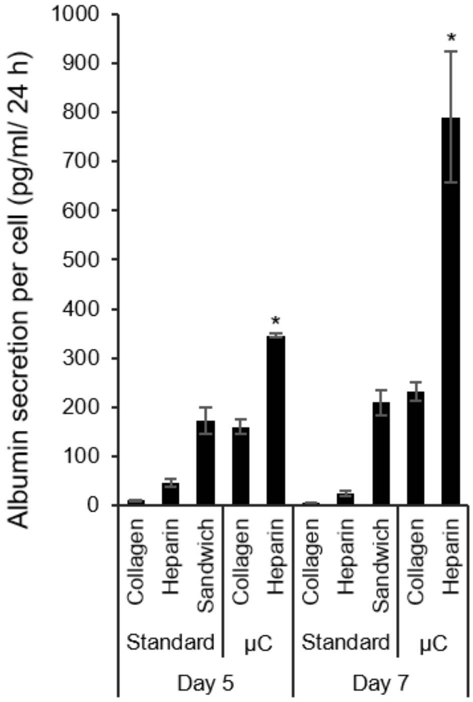

Fig. S6. Effect of heparin hydrogel. ELISA analysis of albumin secretion by hepatocytes at 5 and 7 days of culture. Hepatocytes were cultured under three different conditions: collagen gel sandwich in standard 12-well plates (col gel sandwich), micro-chambers (µCs)-coated with monomeric type I collagen (col type I), and µCs-coated with hepatin gel (Hep gel).

Heparin gel was pre-incubated with collagen type I for cell attachment. Error bars indicate standard deviation (SD) of the mean for n = 3 samples. *:$p < 0.05$.

Supplementary Tables

Table S1. Main parameters for modeling of HGF secretion.

**Parameters**	**Values**
Diffusivity of HGF (D_{HGF})	8.5×10^{-7} cm^2 / s [4]
HGF secretion rate (σ_{sec})	3.121×10^{-3} pg /48 hr per cell
Cell density	µC: 1.5×10^4 cells / device<br>12-well plate: 1.0×10^5 cells / well
Media (volume)	µC: 500 µL / device<br>12-well plate: 1 mL / well

Table S2. Main parameters for modeling of glucose supply.

**Parameters**	**Values**
Initial concentration of glucose	4500 mg / L
Diffusivity of glucose ($D_{glucose}$)	8.33×10^{-6} cm^2 / s [5]
Glucose consumption rate (σ_{con})	8×10^{-9} nmol / s / cell [2]
Cell density	µC: 1.5×10^4 cells / device<br>12-well plate: 1.0×10^5 cells / well
Media (volume)	µC: 500 µL / device<br>12-well plate: 1 mL / well

Table S3. Parameters for oxygen tension modeling.

Parameters	Values
Oxygen Consumption rate (V_{max})	0.38 $nmol/s$ /10^6 hepatocytes [1]
Diffusion Coefficient of Oxygen in PDMS	$7.88 \times 10^{-5}\ cm^2/s$ [3]
Diffusion Coefficient of Oxygen in media	$2.8 \times 10^{-5}\ cm^2/s$ [3]
Diffusion Coefficient of Oxygen in cell	$9.5 \times 10^{-6}\ cm^2/s$ [3]
Oxygen Solubility in PDMS	1.25 mM/atm [3]
Oxygen Solubility in culture media	0.22 mM/atm [3]
Oxygen Solubility in cell	1.049 mM/atm [3]
Cell density inside microchamber	1.5×10^4 cells / device
Michaelis constant (K_m)	5.6 mmHg [1]
Media volume	500 µL / device

Table S4. Oxygen level in different culture systems.

Culture system	Media height	Oxygen level (mmHg) at 24 h
PDMS-on-glass microchamber	75 µm	127.13
	1 mm	98.45
All PDMS microchamber	75 µm	144.01
	1 mm	137.16
Standard 12-well dish (glass slide placed in each well)	2 mm	51.69

Table S5: Primer sequences used for quantitative real-time PCR.

Albumin-F	CAT CCT GAA CCG TCT GTG TG
Albumin-R	TTT CCA CCA AGG ACC CAC TA
HGF-F	CTT CTG CCG GTC CTG TTG
HGF-R	TCT TCT CTT CTT CTG TCC TTC TGC
Igf1-F	GCA TTG TGG ATG AGT GTT GCT
Igf1-R	CAG CGG ACA CAG TAC ATC TCC
Col1a1-F	CAT GTT CAG CTT TGT GGA CCT
Col1a1-R	GCA GCT GAC TTC AGG GAT GT
TGF-β-F	CCT GGA AAG GGC TCA ACA C
TGF-β-R	CAG TTC TTC TCT GTG GAG CTG A
TAT-F	GGT CGC TTC TTA CTA CCA CTG TC
TAT-R	CCG CTT GTC AGA ATG ACA TC
E-cadherin-F	CGT GGA TGT GGT AGA CGT GAA
E-cadherin-R	TTC TTC GCA GGC ACA AAA AT
G6P-F	TCT GTC CCG GAT CTA CCT TG
G6P-R	GTA GAA TCC AAG CGC GAA AC
EGF-F	TGC CTT GCC CTG ACT CTA C
EGF-R	AGC CAA TGA CAC AGT TGC AC
TGFα-F	TCA GTA TCG GGC ATC CAT GTT
TGFα-R	CCA TCC CCA CAG CCT TAC TTT
BMP7-F	GGC TGG CAG GAC TGG ATC AT
BMP7-R	ACC AGT GTC TGG ACG ATA GC
FGF7-F	CTG CTC TAT ATG CGC AAA TGG
FGF7-R	GAG GTG GAA GCA CGG TCT GT
CTGF-F	GGC AGG GCC AAC CAC TGT GC
CTGF-R	CAG TGC ACT TGC CTG GAT GG
CYP1A2-F	ACC ATC CCC CAC AGT ACA A
CYP1A2-R	GTT GAC CTG CCA CTG GTT TA
GAPDH-F	AGA CAG CCG CAT CTT CTT GT
GAPDH-R	CTT GCC GTG GGT AGA GTC AT

References

[1] Cho CH, Park J, Nagrath D, Tilles AW, Berthiaume F, Toner M, et al. Oxygen uptake rates and liver-specific functions of hepatocyte and 3T3 fibroblast co-cultures. Biotechnol Bioeng. 2007;97:188-99.

[2] Nyberg SL, Remmel RP, Mann HJ, Peshwa MV, Hu WS, Cerra FB. Primary hepatocytes outperform Hep G2 cells as the source of biotransformation functions in a bioartificial liver. Ann Surg. 1994;220:59-67.

[3] Kim MC, Lam RHW, Thorsen T, Asada HH. Mathematical analysis of oxygen transfer through polydimethylsiloxane membrane between double layers of cell culture channel and gas chamber in microfluidic oxygenator. Microfluid Nanofluidics. 2013;15:285-96.

[4] Young ME, Carroad PA, Bell RL. Estimation of Diffusion-Coefficients of Proteins. Biotechnol Bioengineer. 1980;22:947-55.

[5] Amsden B. Solute Diffusion within Hydrogels. Mechanisms and Models. Macromolecules. 1998;31:8382–95.
